# Prehospital emergency anesthesia: A single-center retrospective analysis of guideline adherence and unexpected incidents

**DOI:** 10.1371/journal.pone.0310146

**Published:** 2024-09-06

**Authors:** Syrina Beierle, Alexander Beierle, Rolf Rossaint, Stefan K. Beckers, Hanna Schröder, Marc Felzen

**Affiliations:** 1 Medical Faculty RWTH Aachen University, Department of Anesthesiology, University Hospital RWTH Aachen, Aachen, Germany; 2 Aachen Institute for Rescue Management & Public Safety, City of Aachen and University Hospital RWTH Aachen, Aachen, Germany; 3 Department of Anesthesiology, Asklepios Klinik Lich GmbH, Lich, Germany; 4 Medical Direction of Aachen Fire Department, Aachen, Germany; Sapienza University of Rome: Universita degli Studi di Roma La Sapienza, ITALY

## Abstract

Although prehospital emergency anesthesia (PHEA), with a specific focus on intubation attempts, is frequently studied in prehospital emergency care, there is a gap in the knowledge on aspects related to adherence to PHEA guidelines. This study investigates adherence to the “Guidelines for Prehospital Emergency Anesthesia in Adults” with regard to the induction of PHEA, including the decision making, rapid sequence induction, preoxygenation, standard monitoring, intubation attempts, adverse events, and administration of appropriate medications and their side effects. This retrospective study examined PHEA interventions from 01/01/2020 to 12/31/2021 in the city of Aachen, Germany. The inclusion criteria were adult patients who met the indication criteria for the PHEA. Data were obtained from emergency medical protocols. A total of 127 patients were included in this study. All the patients met the PHEA indication criteria. Despite having a valid indication, 29 patients did not receive the PHEA. 98 patients were endotracheally intubated. For these patients, monitoring had conformed to the guidelines. The medications were used according to the guidelines. A significant increase in oxygen saturation was reported after anesthesia induction (p < 0.001). The patients were successfully intubated endotracheally on the third attempt. Guideline adherence was maintained in terms of execution of PHEA, rapid sequence induction, preoxygenation, monitoring, selection, and administration of relevant medications. Emergency physicians demonstrated the capacity to effectively respond to cardiorespiratory events. Further investigations are needed on the group of patients who did not receive PHEA despite meeting the criteria. The underlying causes of decision making in these cases need to be evaluated in the future.

## Introduction

A central role of emergency medicine is to secure the airway and ensure the best possible oxygenation and ventilation for emergency patients in an out-of-hospital setting [1]. However, previous studies indicate that the administration of prehospital emergency anesthesia (PHEA) represents a rare exceptional situation for all involved parties [2]. Even physicians with anesthesiologic experience find themselves working in an ad-hoc team that they are not familiar with or trained in, and that they cannot rely on the routines and resources of a hospital environment, including the presence of an anesthesia nurse [3]. Nevertheless, it was demonstrated that specific guidelines, team training, and revised drug systems improved patient safety during PHEA, whereas the indications for PHEA remained largely unchanged [4–6].

Inadequate execution of PHEA is associated with heightened risk of adverse events, morbidity, and mortality [7,8]. Previous studies in out-of-hospital settings have reported a wide range of incidents, such as hypoxia and hypotension, during the administration of the PHEA [9]. This is highly relevant because the quality of execution depends on various factors including specific guidelines, standardized techniques, medical specialties, and physicians’ prior training [10,11]. Moreover, incorrectly performed PHEA can lead to multiple adverse events that threaten patient safety and outcomes such as hypoxia, hypotension, arrhythmias, circulatory arrest, and ultimately death [12,13]. Therefore, proper and quality-assured execution of the PHEA in a prehospital setting is indispensable.

Several studies have examined the quality of the PHEA and associated incidents, mostly focusing on intubation success rates [14,15]. These studies highlighted the differences in quality based on the qualifications of the physicians and variations among different countries [16–18]. However, comprehensive studies on the quality of PHEA induction and its compliance with guidelines have rarely been published. Specifically, the effectiveness of a system exclusively operated by experienced emergency medical service (EMS) physicians specialized in anesthesiology is unclear.

This study aimed to examine the adherence of PHEA to the German “Guidelines for Prehospital Emergency Anesthesia in Adults” [19], administered by EMS physicians with anesthesiologic experience in Aachen, Germany. In addition to the indication for PHEA, the following aspects were investigated as primary outcomes: Rapid sequence induction, preoxygenation, standard monitoring, endotracheal intubation attempts, hypoxia, hypotension, and tachycardia. The secondary outcomes included type of medications used (propofol, midazolam, thiopental, fentanyl, ketamine, succinylcholine, and vecuronium) and related adverse effects. All these primary and secondary outcomes were examined to thoroughly assess guideline adherence.

## Materials and methods

### Study design

This was a retrospective observational study conducted from 01/01/2020 to 12/31/2021 involving the EMS in Aachen, Germany. The data used in this study were obtained on 11/23/2022.

### EMS system in Aachen, Germany

The EMS in Germany is similar to that in other European countries and is characterized by ambulances staffed with paramedics and other vehicles manned by an EMS physician. These operatives have built a rendezvous system. A dispatching center operator decides which resources to alert based on the incoming emergency. In cases of potentially life-threatening emergencies, both units are alerted [20,21]. According to the Statistical Yearbook 2020–2021 of Aachen, in 2020 and 2021, Aachen had approximately 258,708 residents, with a population density of 1,609 people per square kilometer; furthermore, 2,672 EMS physician interventions were reported. Two vehicles manned by an EMS physician are placed 24 / 7 on site. In contrast to many other cities in Germany, in Aachen, all EMS physicians have experience in the field of anesthesia and must have at least 1 year of experience in intensive care, be in at least the fourth year of their specialist training in anesthesiology, have an 80-hour certificate course in emergency medicine, and pass an examination held by the Medical Association [22]. Intubation and PHEA can be conducted only in the presence of an EMS physician in Aachen. Therefore, the focus of this study was on emergencies that require vehicles manned by an EMS physician.

### Inclusion and exclusion criteria and data collection

**Fig 1:**
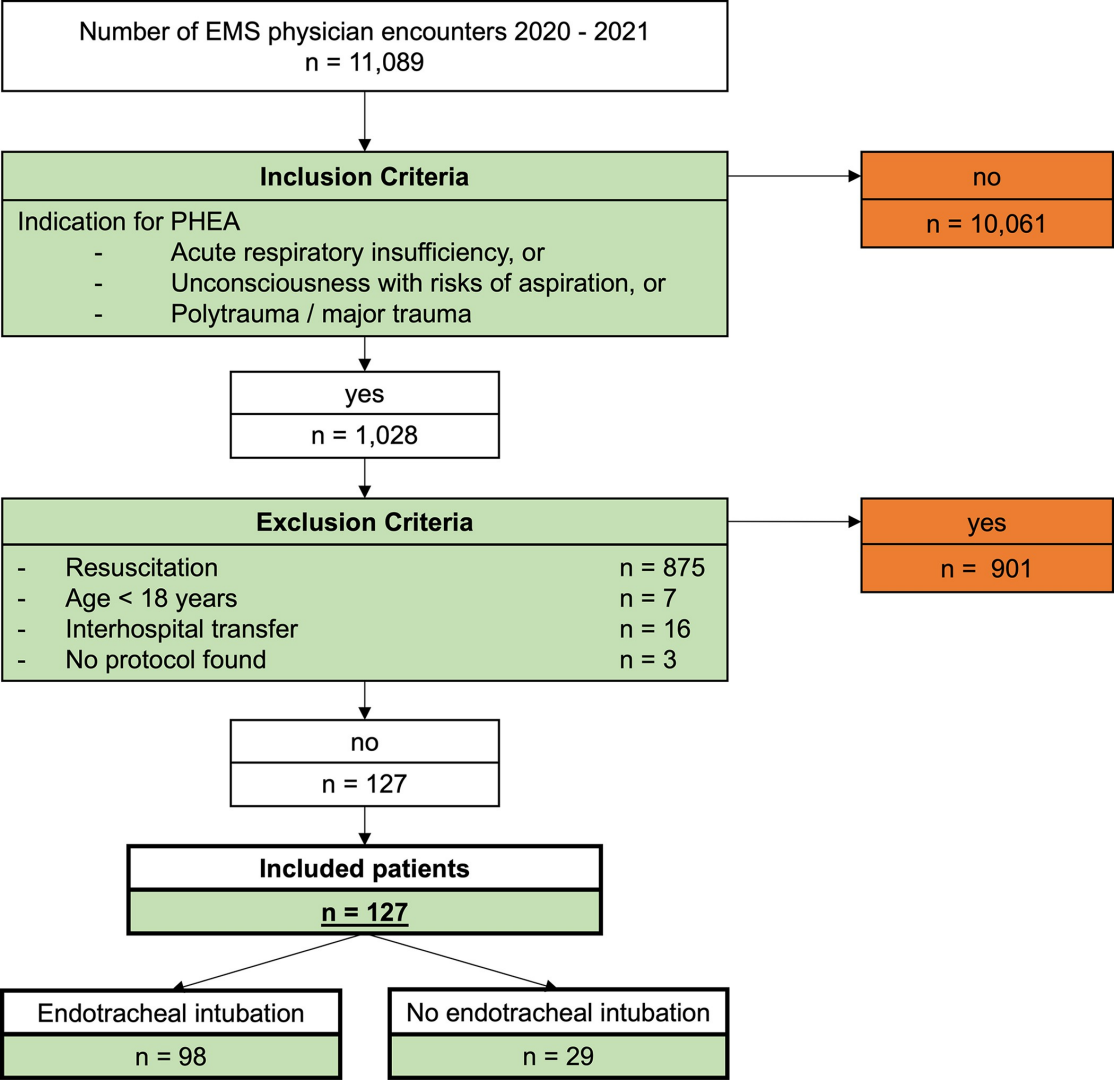
Inclusion and exclusion criteria for the study sample. PHEA, prehospital emergency anesthesia; EMS, emergency medical service.

The inclusion and exclusion criteria are shown in Fig 1.

First, considering the inclusion criteria, patients who fulfilled the indications for PHEA were included. Second, patients who were resuscitated were excluded because the resuscitation procedure followed a separate guideline and was not entirely comparable to other PHEA scenarios. This resulted in patients with return of spontaneous circulation also not being included. Furthermore, the exclusion criteria were age < 18 years, inter-hospital transfers, and no protocol. These criteria result in a study group of 127 patients. These 127 patients were divided into one group of 98 patients who received a PHEA and another group of 29 patients who met the indication for PHEA but not received one.

Data were obtained from standardized, handwritten minimal dataset “MIND3” protocols of the EMS physicians. The minimal dataset in German EMS “MIND3” protocols comprises a specific quantity of signs and sign descriptions authorized by the DIVI (German interdisciplinary association for intensive care and emergency medicine), which are indispensable for documenting prehospital EMS operations performed by EMS physicians [23]. Relevant information from handwritten protocols was digitized to be used in this study. Plausibility checks were applied to minimize the risk of obvious errors in the documentation and digitization.

### Guidelines for prehospital emergency anesthesia in adults

**Fig 2:**
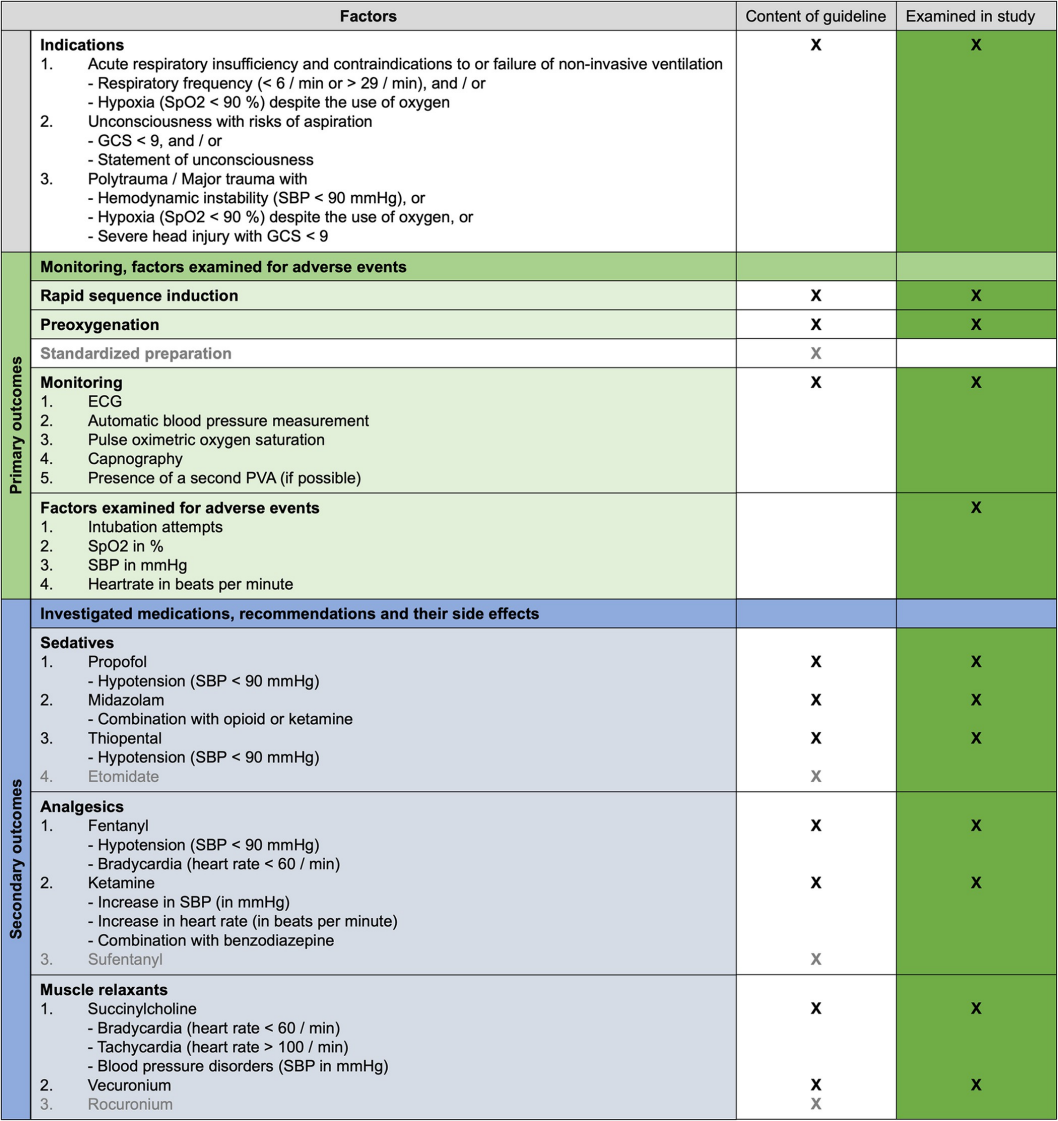
Factors included the guideline and factors investigated in this study. ECG, electrocardiogram; GCS, Glasgow Coma Scale; PVA, peripheral venous access; SBP, systolic blood pressure; SpO2, peripheral oxygen saturation.

The investigations in this study were conducted in light of the “Guidelines for Prehospital Emergency Anesthesia in Adults“, published by the “Prehospital Emergency Anesthesia” working group of the Scientific Working Group Emergency Medicine of the German Society of Anesthesiology and Intensive Care Medicine, which is currently valid in Germany [19]. Fig 2 summarizes the factors included in the guideline and those examined in this study.

Owing to the retrospective study design that used data from written mission protocols, the recommendations for standardized preparation cannot fully be examined, as this was not recorded as standardized data. Furthermore, the guideline provides recommendations on the use and side effects of medications. The use of medications and side effects were explicitly investigated and is presented for each medication based on the drug inventory of the EMS in Aachen. The guideline offers concepts for common emergency situations that have been addressed in this study.

### Examined factors and primary and secondary outcome variables

The patient demographics included age, sex, National Advisory Committee for Aeronautics (NACA) score, and the diagnosis category. The NACA score is widely employed for evaluating medical emergencies in numerous European nations and has seven classifications: NACA 1 –minor disturbance, NACA 2 –moderate disturbance, NACA 3 –severe but not life-threatening disorder, NACA 4 –potentially life-threatening, NACA 5 –acute risk of death, NACA 6 –cardiac arrest, NACA 7 –death [21]. To categorize the diagnosis, synonyms for diseases and similar conditions were clustered into the following seven categories based on the German resuscitation registry and the guidelines for PHEA in Aachen and Germany: trauma, intracerebral bleeding / stroke, seizure, intoxication, respiratory insufficiency, cardial disease, and others. In addition, the criteria for the indication of PHEA were highlighted. The indications for PHEA were reviewed according to the guidelines, consisting of three aspects: acute respiratory insufficiency, assessed as respiratory frequency (< 6 / min or > 29 / min), hypoxia (peripheral oxygen saturation (SpO_2_) < 90% despite oxygenation attempts), and contraindications to / failure of non-invasive ventilation [24]. Second, unconsciousness with risks of aspiration was determined using the Glasgow Coma Score (GCS) < 9 and the statement of unconsciousness [25]. Third, polytrauma / major trauma was defined as hemodynamic instability with systolic blood pressure (SBP) < 90 mmHg [26], hypoxia with SpO_2_ < 90% despite the use of oxygen, or severe head injury with a GCS < 9 [27].

To examine the key recommendations of the guideline as **primary outcome** variables, rapid sequence induction, preoxygenation, standard monitoring, and adverse events were reviewed (Fig 2). The occurrence of the following adverse events was measured in absolute numbers and, if applicable, in units of vital signs: first-pass success rate and intubation attempts aimed at endotracheal intubation, hypoxia (SpO2 < 90%), hypotension (SBP < 90 mmHg), and heart rate (beats per minute). For this purpose, the initial values at the scene were compared with the lowest / highest values recorded after successful anesthesia induction and intubation as well as with the last recorded values upon arriving at the hospital.

Prehospital EMS-specific guidelines on medications such as sedatives, analgesics, and neuromuscular blocking agents, and their characteristics as well as side effects were used to examine the quality of the PHEA procedures performed as secondary outcomes. We did not consider the following medications, as they are not stocked for EMS in Aachen: Etomidate, sufentanyl, and rocuronium. Fig 2 outlines the medications that were examined.

### Statistical analysis

Plausibility checks of the protocol data were conducted to reduce and correct obvious mistakes in documentation before statistical analysis. In the individual analyses, we excluded patients for whom relevant data were missing and not reproducible. Statistical analyses were conducted by using Stata (StataCorp LLC, Texas, USA, 2016, version 13.1) and Excel (Microsoft, Washington, USA, 2023, Microsoft 365, version 16.75.2) software. We considered two-sided p values < 0.01 significant, applying the exact test according to Fisher, Pearson’s chi-squared test, and paired t-tests.

### Ethics approval and consent to participate

The Ethics Committee at the RWTH Aachen Faculty of Medicine (Pauwelsstraße 30, 52074 Aachen, Germany) reviewed the analyses and found no constraints (Approval number: 180/18). The requirement for obtaining informed consent was waived by the Ethics Committee at the RWTH Aachen Faculty of Medicine (Pauwelsstraße 30, 52074 Aachen, Germany; Head: Prof. Hausmann), the Center for Translational and Clinical Research (CTC-A) of RWTH Aachen University, and the relevant data protection officers. Given that this retrospective analysis was conducted anonymously as part of the legally mandated quality assurance responsibilities of municipal authorities. Access to the data for this study was obtained on 11/23/2022, and data processing was conducted using a pseudonymized dataset.

## Results

### Demographics of the study population

In the 2-year study period of 2020–2021, vehicles manned with an EMS physician were alerted by the dispatch center 11,089 times. In total, 127 patients were included in this study. Therefore, only 1.1% (127 / 11,089) of all emergency patients with physician encounters were examined. The patients had a mean age of 62.11 (standard deviation (SD) 20.40) years; 37.0% (47 / 127) of the patients were female. Out of all patients with indication for PHEA 77.2% (98/127) received PHEA and endotracheal intubation, 22.8% (29/127) did not receive PHEA. The patients’ characteristics, the training level of the EMS physicians, and the frequency of diagnosis groups are presented in Table 1.

**Table 1:** Patient characteristics, level of training of EMS physicians, and diagnoses.

	Endotracheal intubation (n = 98)	No endotracheal intubation (n = 29)
Age, mean (standard deviation (SD))	61.42 (20.76)	64.69 (19.20)
Sex female, n (%)	41 (41.8)	6 (20.7)
Specialist, n (%)	80 (81.6)	18 (62.1)
Resident in the 4^th^, 5^th^, or 6^th^ year, n (%)	18 (18.4)	11 (37.9)
**Category of diagnoses**		
Intracerebral bleeding / Stroke, n (%)	34 (34.7)	4 (13.8)
Respiratory insufficiency, n (%)	19 (19.4)	10 (34.5)
Trauma, n (%)	16 (16.3)	5 (17.2)
Seizure, n (%)	13 (13.3)	5 (17.2)
Intoxication, n (%)	7 (7.1)	3 (10.3)
Others, n (%)	6 (6.1)	0 (0.0)
Cardial disease, n (%)	3 (3.1)	2 (6.9)

Others: Anaphylaxis, meningitis, burns, sepsis, and acute abdomen.

**Fig 3:**
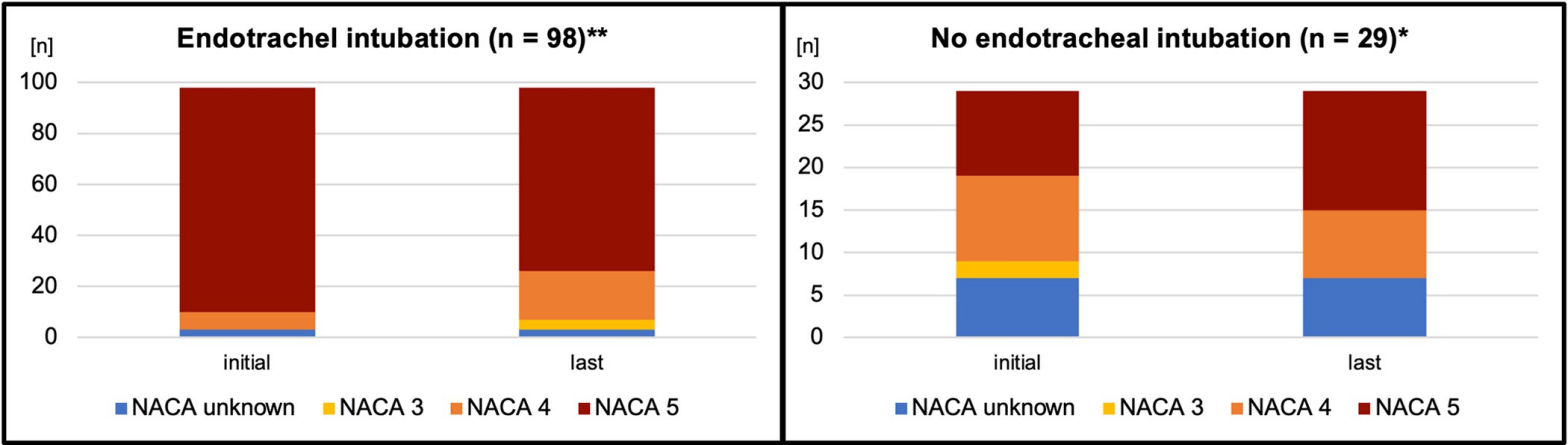
NACA score. NACA, National Advisory Committee for Aeronautics; * represents significant difference (p < 0.01); ** represents significant difference (p < 0.001).

Fig 3 outlines the NACA score.

### Indications for PHEA and primary outcomes

**Fig 4:**
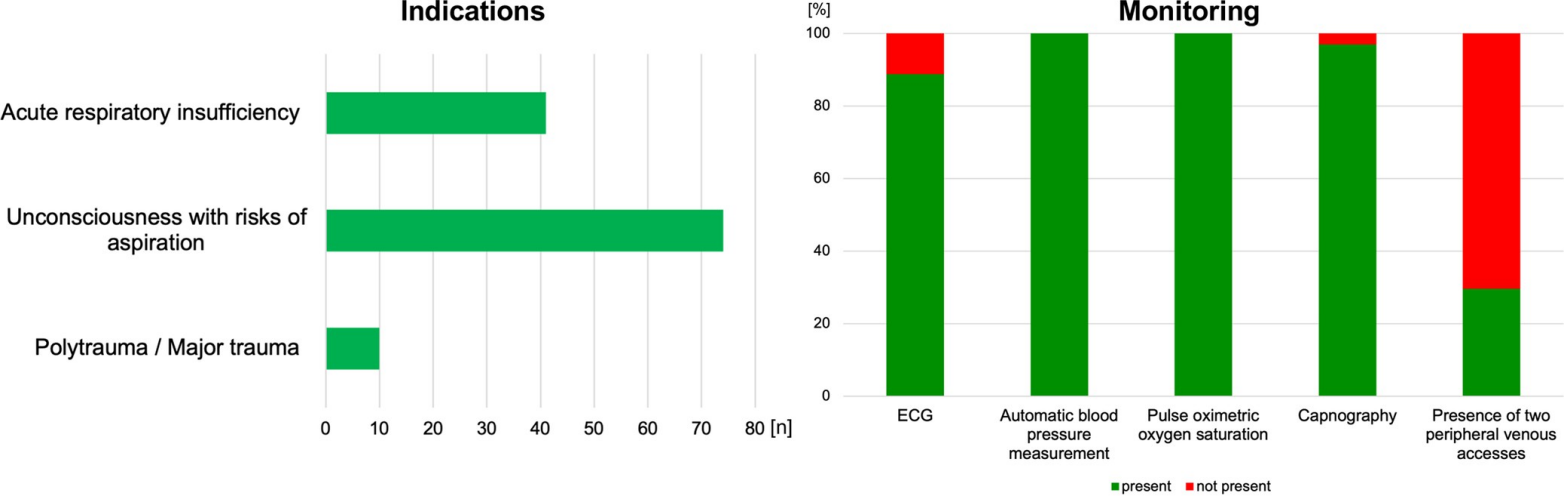
Indications and monitoring. ECG, electrocardiogram.

We identified 127 patients with indication for PHEA. Fig 4 summarizes the overall indications and rates of monitoring of the patients who were intubated.

Some patients had acute respiratory insufficiency, and unconsciousness, with risks for aspiration. Moreover, we screened all 11,089 emergency patients in 2020 and 2021 to determine if they met the indications for PHEA, according to the guidelines. We identified 22.8% (29 / 127) of the patients (age > 18 years), who should have received PHEA: 44.8% (13 / 29) patients with an indication of acute respiratory insufficiency, 41.4% (12 / 29) patients with an indication of unconsciousness with risks of aspiration, and 17.2% (5 / 29) patients with an indication of trauma. 3.5% (1 / 29) of the patients met the criteria of acute respiratory insufficiency and trauma.

The following analyzes refer to the study group of the intubated patients (n = 98). Since the patients with a clear indication who did not receive a PHEA (n = 29) were not treated according to the guideline being studied, these analyses are not applicable to that group.

**Fig 5:**
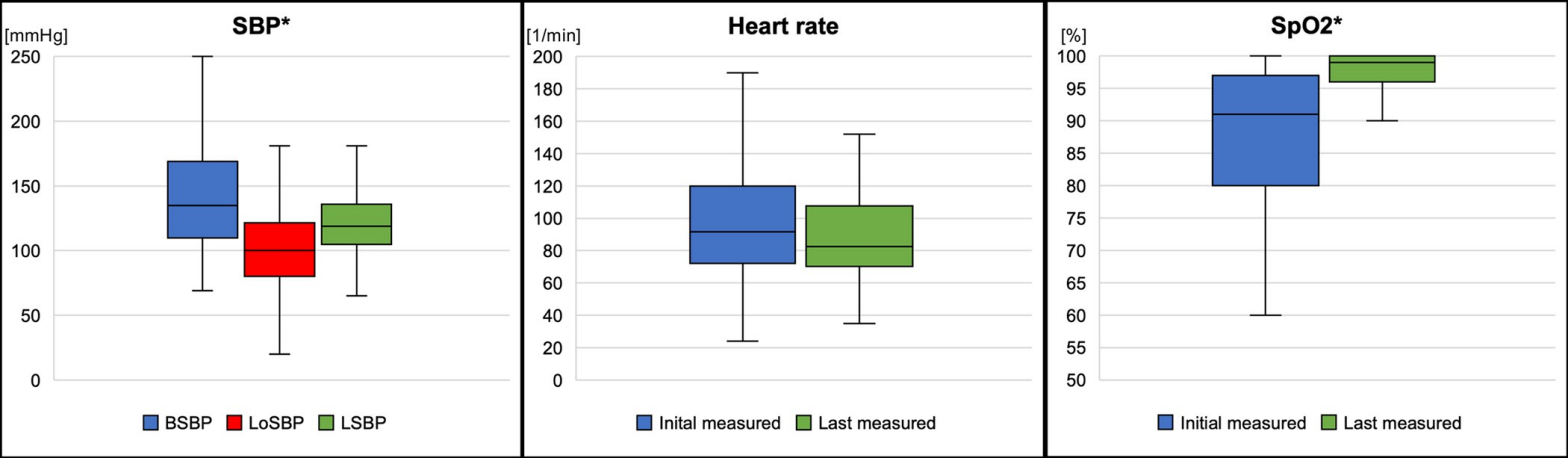
Adverse events in SBP, heart rate, and SpO2. SBP, systolic blood pressure; BSBP, beginning systolic blood pressure; LoSBP, lowest systolic blood pressure after intubation; LSBP, last measured systolic blood pressure; SpO2, peripheral oxygen saturation; * represents significant difference (p < 0.001).

All patients were preoxygenated and underwent rapid sequence induction, which involves the use of a video laryngoscope. Considering the completeness of vital sign monitoring, Fig 4 summarizes the results. There was no significant lack of monitoring of the guidelines for these items. In 29.6% (29 / 98) cases, the patients received more than one peripheral venous access (PVA). Successful endotracheal intubation was achieved with a first-pass success rate of 94.9% (93 / 98); 99.0% (97 / 98) of the patients were successfully endotracheally intubated after the second attempt. After three attempts, all intubation procedures were successful (98 / 98). Moreover, we compared the initial measured SBP, lowest SBP after intubation, and last SBP measured during the mission. Furthermore, we examined the initial and final heart rates and oxygen saturation levels (Fig 5).

The difference between the SBP measured at the beginning of the emergency deployment (BSBP) and the lowest measured after intubation (LoSBP) was significant (p < 0.001; Confidence interval (CI): -42.62 to -29.10). Similarly, the difference between the LoSBP and the last measured value (LSBP) was significant (p < 0.001; CI: 12.11 to 21.98), as was the difference between the initially measured and the last measured value (p < 0.001; CI: -26.4 to -11.23). No significant difference was observed in the heart rate between the initial and last measured values (p = 0.02). In contrast, concerning the oxygen saturation, a significant improvement between the first and last measured values was found (p < 0.001; CI: 8.12 to 14.56).

### Secondary outcomes

**Fig 6:**
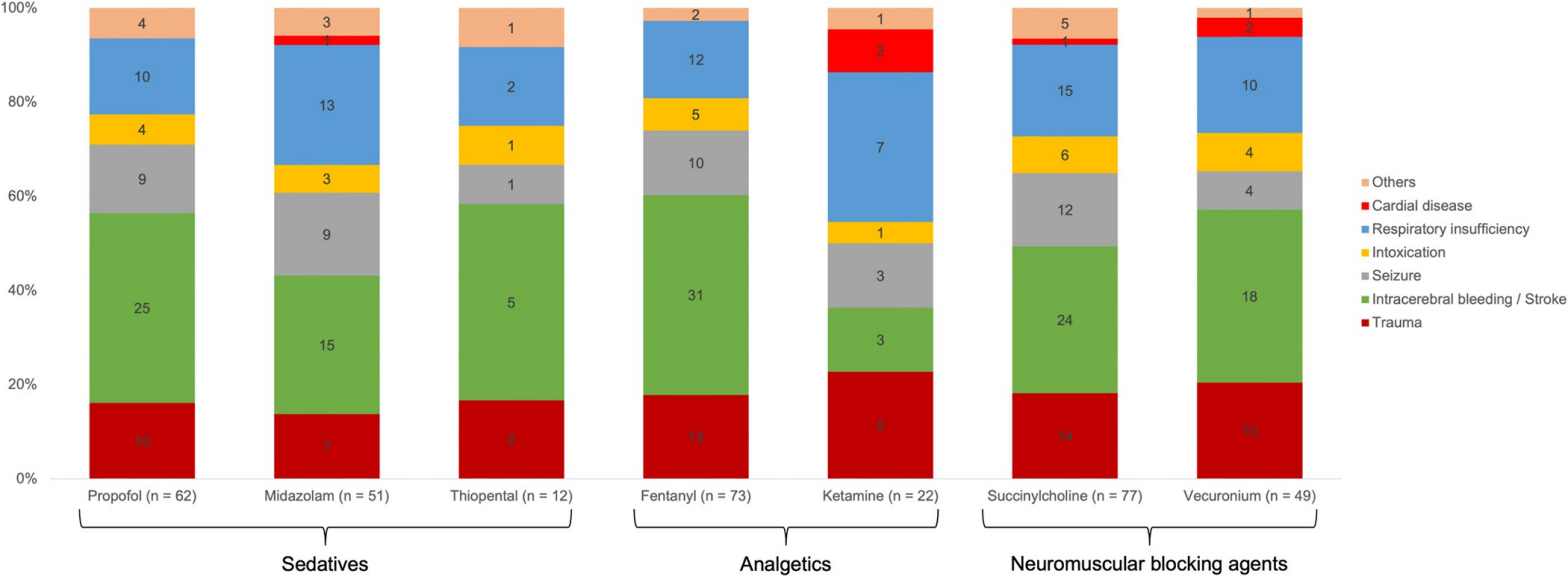
Medications and their use for specific diagnose groups.

To examine the medications and their associated adverse events, we divided them into sedatives, analgesics, and neuromuscular blocking agents. Fig 6 outlines the use of these medications in the specific diagnosis groups.

**Fig 7:**
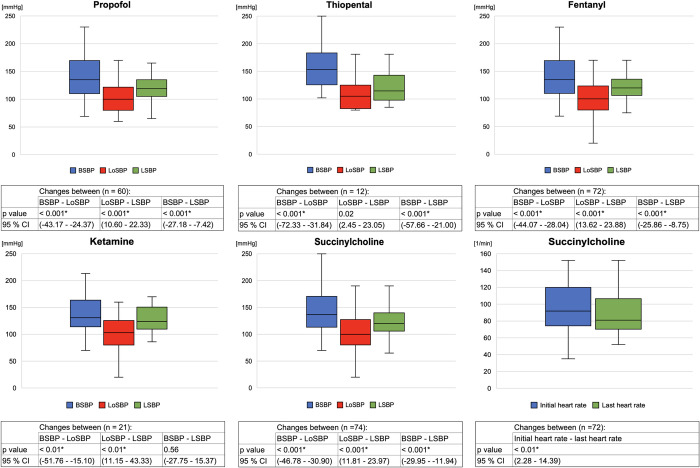
SBPs and heart rate associated with each medication. BSBP, beginning systolic blood pressure; CI, Confidence interval; LoSBP, lowest systolic blood pressure after intubation; LSBP, last measured systolic blood pressure; * represents significant difference.

The following medication combinations were the most used ones: propofol, fentanyl, and succinylcholine (45 / 98), and propofol, fentanyl, and vecuronium (28 / 98). Owing to the retrospective nature of the data collection, we are unable to draw precise conclusions regarding the exact timing of medication combinations. Therefore, in the following analysis, we examined individual medication administration and its effects to avoid introducing bias. The association of medication with changes in vital parameters was determined using Fisher’s exact test and Pearson’s chi-squared test, the association of the medication with changes in vital parameters will be highlighted. Fig 7 outlines the side effects of the medications used.

First, the administered sedatives and their side effects were examined. After a significant decrease in SBP following propofol administration 61.3% (38 / 62) of patients received vasoactive substances. Considering midazolam, there was no significant association between the administration of midazolam and opioids or ketamine (p = 0.06). We could not determine a significant relationship between thiopental use and SBP < 90 mmHg.

Second, the administered analgesics and their side effects were analyzed. Taking fentanyl into account, after the significant decrease in SBP 65.8% (48 / 73) of patients received vasoactive substances. The relationship between fentanyl administration and SBP < 90 mmHg was not significant (p = 0.47). The heart rate did not change significantly between the initial final measured values (p = 0.02). No significant relationship was found between fentanyl administration and heart rate < 60 / min (p = 0.15). Regarding ketamine, the analyses have demonstrated no significant difference between the initially recorded heart rate and the heart rate measured at the end (p = 0.51). A significant relationship was found between the ketamine and benzodiazepine administration in 86.4% of the patients (p < 0.001).

Finally, adverse effects of neuromuscular blocking agents were investigated. With regard to succinylcholine, after a significant decrease in SBP, 54.6% (42 / 77) of the patients received vasoactive substances. No significant relationship between succinylcholine administration and bradycardia (p = 0.05) or tachycardia (p = 0.50) was observed. Vecuronium was administered for 50.0% (49 / 98) of the patients. No adverse events were observed during vecuronium administration. The SBPs associated with each medication that demonstrated a significant reduction as an adverse effect are depicted in Fig 7.

## Discussion

We examined the data of patients who required endotracheal intubation and underwent PHEA to determine the level of guideline adherence, incidents, and treatment outcomes. The data indicated that there are various incidents that vary depending on the diagnosis category and medications used and are addressed by different means. Overall, the adverse effects as well as the NACA scores demonstrated that the patient group was critically ill and presented a challenge to prehospital treatment by the EMS. There is a noticeable difference between intubated and non-intubated patients. The NACA score for intubated patients shows a significant decrease from the start to the end of the intervention, suggesting that these patients achieved an improved condition as a result of the intervention. Conversely, in non-intubated patients, the NACA score exhibits a significant increase from the beginning to the end, as these patients did not receive the intervention of PHEA and intubation. As the NACA score only represents a severity scoring system, we cannot conclude on patients’ clinical conditions at hospital handover.

All 127 patients fulfilled at least one indication for PHEA; therefore, no patient without a clear indication for PHEA received it. 29 patients were identified as having a clear indication but did not receive PHEA. Three patients did not undergo capnography, although it was mandatory. In the context of medication administration, well-known side effects occur frequently and must be managed appropriately. What led to the deviations from the guideline? Studies have indicated that adherence to checklists, guidelines, and predefined standards improves patient safety [4,15,28,29]. The paternalistic model of “the doctor knows best” has mostly been replaced by guideline-based medicine, which is built on expert consensus [29]. The PHEA guideline recommends indications and processes that provide the highest possible standards for patient safety. In recent years, significant progress has been made in the practical care of patients who require a PHEA. Procedures and checklists have been developed to improve patient safety. Nevertheless, the relevance of indications has remained almost unchanged [6]. Intubation of critically ill patients in an in-hospital setting is positively influenced by the training and experience of the physician and the selected equipment [6,30]. In contrast, in a prehospital setting, various factors contribute to decision-making, and the people involved in a prehospital setting may not had access to hospital resources [2,3]. Although the respective physicians are requested to follow existing guidelines, the decision-making process is multifactorial and depends on various aspects such as experience and proficiency in certain techniques, transportation time to the hospital, or instability of the patient. Additionally, there are other guidelines, such as the guideline for polytrauma patients, which provides recommendations and applies in individual cases of each patient. Each situation is unique in its individual circumstances. Future studies should examine these aspects, namely the decision-making processes for and against PHEA, combination of different medications, and level of training of the participating EMS physicians. Additionally, the results show incidents and adverse events. This could indicate that the guideline should be adjusted and further developed in the future.

### Indications for PHEA and primary outcomes

Indications for prehospital interventions have not undergone significant changes in recent years. The most common indications for PHEA include airway and breathing challenges [6]. Therefore, the high proportion of patients in our study who had acute respiratory insufficiency and unconsciousness with risks for aspiration demonstrates the relevance of securing the airway in prehospital emergency care. Additionally, 10.2% (10 / 98) of the intubated patients had an indication of trauma and received PHEA. Thus, our data aligns with those reported in other studies [6,31].

When considering patients who met the indications for PHEA according to the guidelines but did not receive PHEA, all three indication groups were represented. During the study period, 22.8% (29 / 127) of the patients treated by an EMS physician did not receive PHEA. However, the most common indications in these cases were acute respiratory insufficiency and unconsciousness with the risk of aspiration. This is roughly similar to the distribution of the PHEA performed during the study period. Furthermore, the patients who did not receive PHEA were on average, 64.69 years old (SD 19.20), similar to the average age (61.42 years, SD 20.76) of the patients who received PHEA. Other studies have shown that performance and quality of emergency anesthesia depend on the medical specialty and experience of the physicians [18]. Interestingly, these studies did not indicate whether this influenced the decision to discontinue emergency anesthesia. In our study, all EMS physicians had a background in anesthesia. Notably, among the administered PHEA, there were more specialists involved (81.6%) than those who did not (62.1%). However, the training duration of the resident physicians showed little variation. In the future, it would be interesting to investigate whether less experienced EMS physicians or other factors, such as short transport distances, contributed to the decision not to perform PHEA despite the indications. These aspects mentioned above could potentially be considered in future revisions of the guideline, incorporating the latest literature.

Most aspects of monitoring were fulfilled. Only 29.6% (29 / 98) of the patients received more than one PVA. The guideline recommends the placement of two peripheral venous cannulas. As our data were collected retrospectively and standardized, we could not ascertain the reasons why more than one PVA was not used. It could be assumed that the EMS physician considered one access to be sufficiently secure.

Further, 3.1% (3 / 98) of the patients did not undergo capnography. Although this aspect is not significant, it can be life-threatening for every patient without it. Capnography is a crucial tool for detecting esophageal intubation and is an extremely important precaution for avoiding and minimizing life-threatening complications during prehospital care [28]. This raises the question of whether this monitoring was omitted from the EMS protocols but was still conducted or whether it was truly not performed despite being an aspect of the instructions in Aachen. This point should be examined in the future.

All patients were successfully endotracheally intubated within a maximum of three attempts. The materials available for securing the airway conformed to the guideline recommendations. This study waives to place specific products in order to avoid conflicts of interest. The success rate of endotracheal intubation varies among personnel with different backgrounds [32]. A study conducted in the Netherlands demonstrated that helicopter EMS physicians achieved a first-pass success rate of 86.9% [33], similar to our first-pass success rate of 94.9% (93 / 98). Furthermore, in a previous study that examined intubation attempts by EMS physicians with an anesthesiologic background, all patients under consideration were successfully endotracheally intubated [34]. This could be attributed to the fact that anesthesiologists are experienced in endotracheal intubation and anesthesia induction [11]. Since our data pertain to physicians with experience in anesthesia, our results are consistent with those of previous studies. Furthermore, our study demonstrated a significant increase in oxygen saturation (p < 0.001), this aligns with a previously described successful intubation aimed at achieving optimal ventilation and oxygenation. Previous studies have shown that oxygen saturation can be ensured and significantly increased in various patient groups through endotracheal intubation [9].

When examining the SBP, a significant decrease was observed after anesthesia induction, followed by a significant increase. In our study, the average SBP decreased by 22.6% and subsequently increased by 16.5%. These results are largely consistent with those of previous studies conducted in out-of-hospital [9] and in-hospital settings [35]. When considering the change in heart rate, it is unlikely that the PHEA caused this. Other events such as blood loss are more probable.

### Secondary outcomes

On examining the administered sedatives, we found that propofol was most frequently used in the intracerebral bleeding / stroke diagnosis group. Because propofol does not cause increased intracranial pressure, its use in the diagnosis of intracerebral bleeding / stroke is well known [19]. Across all diagnosis groups, propofol use was associated with a significant decrease in SBP. This is a well-known side effect [36,37], and the EMS physicians respond by administering medications that significantly increased SBP (e.g., noradrenaline). Our findings are consistent with results of existing studies on in-hospital incidents related to the administration of propofol [36]. The barbiturate thiopental was only used in 12 patients. Thiopental is characterized by its rapid onset of action and ability to reduce intracranial pressure [38]. This may explain its relatively frequent use in patients with intracerebral bleeding / stroke or trauma. However, thiopental can lead to a decrease in blood pressure, which was significant in our study (p < 0.001). Therefore, our findings are consistent with the previously described side effects of thiopental [38,39]. Although there was a significant decrease in SBP, no significant correlation was found between the drop below 90 mmHg and the administration of thiopental. However, the low number of patients who received thiopental must be considered in the interpretation. Additionally, the data indicated a significant increase in SBP after the previously observed drop, indicating that the side effect was effectively managed by the attending EMS physicians.

Among analgesics, ketamine was most administered to patients with respiratory insufficiency and trauma. A unique feature of ketamine is that, depending on the dosage, it can be used for analgesia with preserved protective reflexes, as well as for complete anesthesia induction. Especially in the case of trauma patients who may be trapped or difficult to reach, analgo-sedation with ketamine and a benzodiazepine is administered. It provides the advantages of preserved spontaneous breathing and cardiovascular stability offered by ketamine. Therefore, the use of ketamine in these patient groups was a focal point and aligned with our data. The potential side effects of ketamine include anxiety disorders agitation. Benzodiazepines were administered to counteract these effects [39]. In our study, 86.4% (19 / 22) of the patients who received ketamine also received benzodiazepines, indicating a significant relationship between the administration of these two medications. When considering SBP, a significant decrease was observed after anesthesia induction, followed by a significant increase, indicating that EMS physicians likely managed the decrease appropriately. Ketamine is known for its less pronounced circulatory depressant effects than the other medications considered in this study. This fact supports our data for two reasons: First, the SBP did not fall below 90 mmHg, and second, the reduction (24.7%) with ketamine was smaller than that with thiopental (33.0%) and fentanyl (26.0%).

Finally, neuromuscular blocking agents were examined. Succinylcholine was the most frequently used relaxant. Previous studies have recommended succinylcholine, mainly owing to its rapid onset of action in the context of PHEA [39,40]. Succinylcholine has numerous side effects. In this study, we focused on those that could be well investigated based on the data. The side effects of bradycardia, tachycardia, and significant changes in heart rate were not observed. Similar results were obtained in a prehospital setting previously [41]. According to recent studies, rocuronium is commonly used as a muscle relaxant. Rocuronium is recommended for minimizing potential incidents during PHEA [5]. As rocuronium has only been available in the EMS of Aachen since October 2022 and our study covered the period from 2020 to 2021, the attending EMS physicians had no opportunity to use this medication in the induction of PHEA. As a result, we were unable to investigate the potential side effects, such as a reduced increase in heart rate [5,19,41].

The guideline also provides recommendations regarding medications that can be administered to specific diagnostic groups. For instance, in patients with intracranial bleeding / stroke, the following medications are recommended for anesthesia induction: thiopental or propofol as sedatives; fentanyl, sufentanyl, or ketamine as analgesics; and rocuronium or succinylcholine as muscle relaxants [19]. This is in line with our data, where 73.5% (25 / 34) of the patients in the described diagnosis group received propofol and 14.7% (5 / 34) received thiopental. Fentanyl was administered to 91.2% (31 / 34), ketamine to 8.8% (3 / 34), and succinylcholine to 70.6% (24 / 34) of the patients. Because sufentanyl and rocuronium are not stocked in the vehicles manned by EMS physicians, adherence to the guideline in this regard could not be verified. Overall, the EMS staff adhered to recommendations regarding when specific medications should be administered for PHEA in certain diagnostic groups.

### Limitations

Compared to other studies that examined PHEA, this study included a relatively small sample size. Consequently, the results may not be generalizable and may not be applicable to other regions with slightly different conditions (e.g., variations in physician training and distinct emergency response systems). Another limitation is that patients who underwent endotracheal intubation and received analgesics and sedatives may have been inadvertently overlooked, despite systematic examination. Additionally, the emergency medical protocols included in the analyses were not exclusively designed for this study; this could have led to an underestimation of complications and adherence to guidelines. Furthermore, this study was retrospective, and the emergency medical protocols analyzed were completed by the respective EMS physicians immediately after the intervention; this may have introduced reporting and recall biases. Moreover, which medications were given in combination within a specific timeframe could not be confirmed; as a result, incidents caused by these combinations could not be investigated. Patients with resuscitation and return of spontaneous circulation were excluded. This patient group should be examined separately in further studies to also assess the implementation of PHEA in this context, as studies have emphasized the necessity of expanding the guidelines to include patients who require emergency anesthesia after return of spontaneous circulation [42].

## Conclusions

Overall, all patients who received PHEA were successfully endotracheally intubated by EMS physicians with experience in anesthesia. Potentially significant hypotensive episodes and other incidents were identified and addressed by the administration of appropriate medications. In summary, high guideline adherence was ensured with regard to execution of PHEA and in the selection and administration of appropriate medications. Adequate responses were observed in patients with cardiorespiratory events. Concerning the indications for PHEA, substantial consistency with the guideline could not be identified. Our finding that some patients were not endotracheally intubated and did not receive PHEA despite having an indication for PHEA needs to be examined in future studies to highlight the quality of guideline adherence in this specific patient group. Furthermore, our results highlight potentials and areas that could be further developed in the respective guideline in the future.

## Supporting information

S1 Dataset Data set used for statistical analysis. (XLSX)
